# Intranasal Delivery of Plasma and Platelet Growth Factors Using PRGF-Endoret System Enhances Neurogenesis in a Mouse Model of Alzheimer’s Disease

**DOI:** 10.1371/journal.pone.0073118

**Published:** 2013-09-19

**Authors:** Eduardo Anitua, Consuelo Pascual, Rocio Pérez-Gonzalez, Desiree Antequera, Sabino Padilla, Gorka Orive, Eva Carro

**Affiliations:** 1 Foundation Eduardo Anitua, Vitória, Spain; 2 Neuroscience Group, Instituto de Investigacion Hospital 12 de Octubre (i+12), Madrid, Spain; 3 Biomedical Research Networking Center in Neurodegenerative Diseases (CIBERNED), Madrid, Spain; 4 NanoBioCel Group, Laboratory of Pharmaceutics, University of the Basque Country, School of Pharmacy, Vitoria-Gasteiz, Spain; 5 Networking Biomedical Research Center on Bioengineering, Biomaterials and Nanomedicine, CIBER-BBN, Vitoria-Gasteiz, Spain; Rosalind Franklin University, United States of America

## Abstract

Neurodegeneration together with a reduction in neurogenesis are cardinal features of Alzheimer’s disease (AD) induced by a combination of toxic amyloid-β peptide (Aβ) and a loss of trophic factor support. Amelioration of these was assessed with diverse neurotrophins in experimental therapeutic approaches. The aim of this study was to investigate whether intranasal delivery of plasma rich in growth factors (PRGF-Endoret), an autologous pool of morphogens and proteins, could enhance hippocampal neurogenesis and reduce neurodegeneration in an amyloid precursor protein/presenilin-1 (APP/PS1) mouse model. Neurotrophic and neuroprotective actions were firstly evident in primary neuronal cultures, where cell proliferation and survival were augmented by Endoret treatment. Translation of these effects *in vivo* was assessed in wild type and APP/PS1 mice, where neurogenesis was evaluated using 5-bromodeoxyuridine (BdrU), doublecortin (DCX), and NeuN immunostaining 5 weeks after Endoret administration. The number of BrdU, DCX, and NeuN positive cell was increased after chronic treatment. The number of degenerating neurons, detected with fluoro Jade-B staining was reduced in Endoret-treated APP/PS1 mice at 5 week after intranasal administration. In conclusion, Endoret was able to activate neuronal progenitor cells, enhancing hippocampal neurogenesis, and to reduce Aβ-induced neurodegeneration in a mouse model of AD.

## Introduction

Alzheimer’s disease (AD) is a progressive neurodegenerative disease and the most prevalent cause of dementia in adults. The hallmarks of the disease are amyloid deposits of aggregated β-amyloid (Aβ) peptides and neurofibrillary tangles, which are intracellular aggregates of hyperphosphorylated tau [1]. Increasing evidences suggest that altered or compromised neurogenesis may contribute to the cognitive impairments and neuronal vulnerability that characterize the disease. Indeed, numerous studies report impaired hippocampal neurogenesis in mouse models exhibiting high levels of Aβ, amyloid deposition [2-4], and neurofibrillary tangles [5].

The adult brain has two stable regions of mitotic activity, the subventricular zone of the lateral ventricle in the frontal cortex and the subgranular zone of the dentate gyrus in the hippocampus [6,7]. Parallel to the diminution in neurogenesis, is the decline of growth factors [8,9]. Remarkably in AD, levels of neurotrophic factors are decreased in patient brains, including insulin like growth factor (IGF-I), brain-derived growth factor (BDNF), and vascular endothelial growth factor (VEGF) among others [10-13].

The technology of plasma rich in growth factors (PRGF-Endoret or formerly Endoret) is a relatively new biological therapy that uses patient’s own proteins and growth factors as therapeutics [14-16]. In fact, it is obtained from patient’s own blood and it consists in a supernatant enriched in plasma and platelet-derived proteins and morphogens. The biological basis of Endoret relies on the concentration of platelets within a defined plasma volume. Platelets are then activated by means of calcium to effectively release the protein content stored within their alpha granules [17]. The latter are full of morphogens including platelet-derived growth factor (PDGF), transforming growth factor beta (TGF-β), VEGF, fibroblast growth factor (FGF), epidermal growth factor (EGF), IGF-I and nerve growth factor (NGF) among others [15,18]. Many of these growth factor factors are known to accelerate cell proliferation and differentiation, promote cell survival and stimulate angiogenesis [19-22].

To test the hypothesis that Endoret also functions as a regenerative therapy with an impact on neurodegeneration in AD, we investigated the efficacy of the pool of plasma and platelet-derived proteins as a neurogenic agent *in vivo* using amyloid precursor protein/presenilin-1 (APP/PS1) mice. One critical problem is the way to deliver growth factors in a localized manner within the brain tissue to avoid undesirable peripheral side effects. The development of a less invasive delivery method for brain uptake may significantly improve the prospects of growth factor clinical uses. Intranasal delivery provides a practical, non-invasive method of bypassing the blood-brain barrier (BBB) to deliver therapeutic agents to the brain. In view of this, we explored the hypothesis that intranasal Endoret treatment can improve neurogenesis and ameliorate neurodegeneration in this mouse model of AD.

## Materials and Methods

### Human samples

Five groups of human subjects were studied: (1) young subjects, (2) elderly non-demented controls, (3) mild cognitive impairment (MCI) patients, and two categories of AD patients; (4) mild, and (5) moderate/severe (Table 1). All the samples were obtained from the Neurology Service of the Hospital Universitario 12 de Octubre (Madrid, Spain), after the approval of the ethics committee from the Hospital Universitario 12 de Octubre (Madrid, Spain). Patients provided written informed consent to participate in this study according to the requirement suggested and then approved by the ethics committee. AD cases were diagnosed with dementia according to the Diagnostic and Statistical Manual of Mental Disorders (DSM)-IV criteria, and NINCDS-ADRDA criteria [23]. No neurological symptoms or signs were recorded in elderly control group.

**Table 1:** Demographic characteristics of patients and controls.

	Mean age	Gender	
		Men	Women
Young subjects	33.4 ± 1.81	5	5
Aged patients	75.5 ± 2.3	7	3
MCI patients	80.25 ± 2.5	3	5
AD patients			
mild	80 ± 1.11	4	9
moderate/severe	80 ± 2.22	1	6

### Plasma rich in growth factors (Endoret)

**Figure 1:**
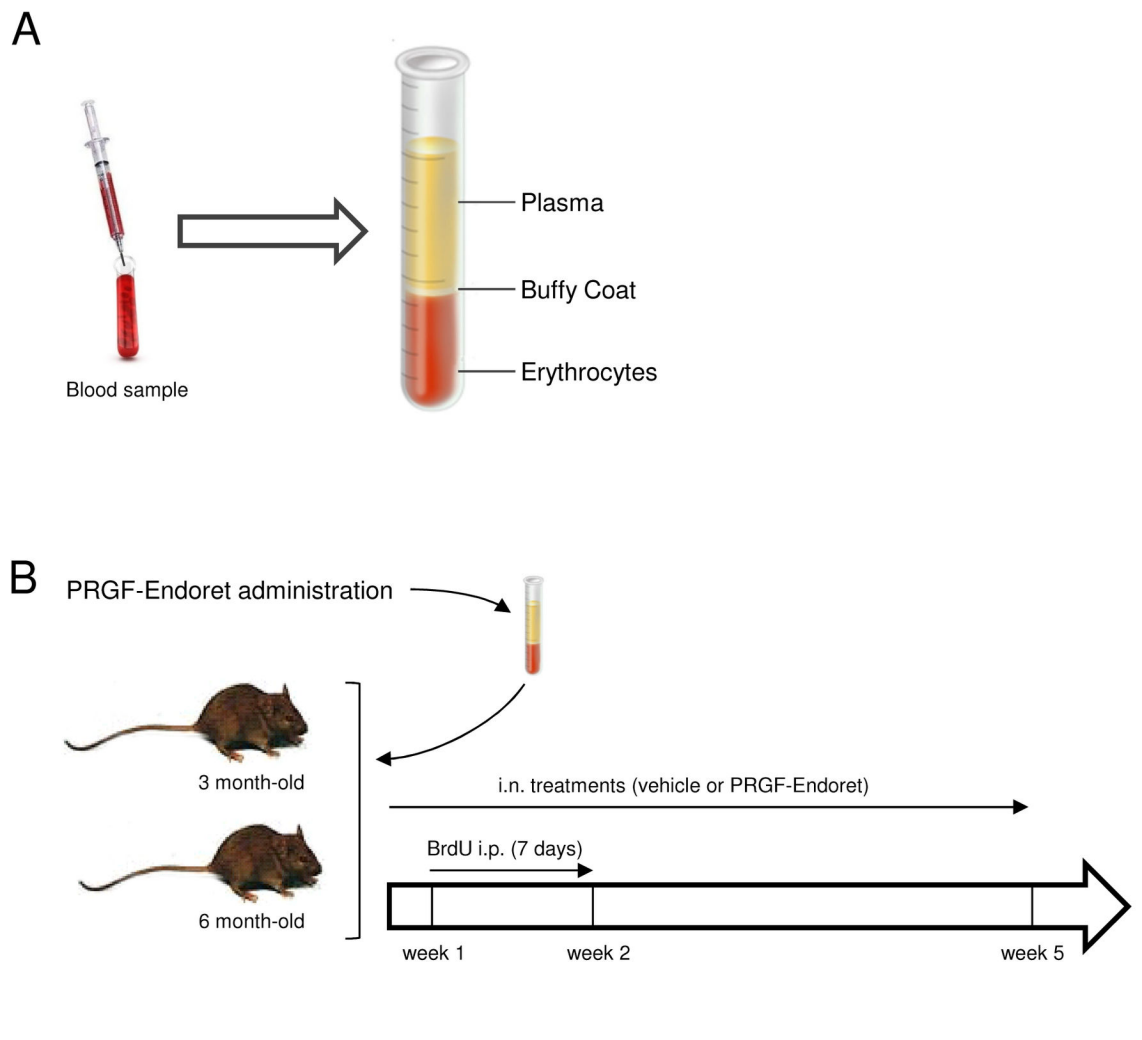
Use of Endoret treatment in a mouse model of AD. A. Scheme of the different plasma fractions obtained with the Endoret technology from blood samples. B. Experimental design for the Endoret treatment in APP/PS1 mice.

Informed consent from all subjects was obtained prior to their participation. Blood samples were obtained through antecubital vein puncture. Plasma rich in growth factors (Endoret) was obtained as follows. Briefly, blood from donor subjects was collected into 9-mL tubes with 3.8% (wt/vol) sodium citrate. Samples were centrifuged at 580*g* for 8 min at room temperature in a PRGF-Endoret system centrifuge (BTI Biotechnology Institute). The plasma fraction containing platelets but not buffy coat and erythrocytes was separated (Figure 1A). Plasma fractions were incubated with calcium chloride (BTI Biotechnology Institute) for 1 h at 37°C in glass tubes. The released supernatants were collected by aspiration after centrifugation at 1000*g* for 20 min at 4°C. Finally, platelet enriched plasma fractions were aliquoted and stored at −80°C until use. Growth factors (TGF-β1, PDGF, VEGF, HGF, EGF, IGF-1, and NGF) were measured in the supernatants using commercially available colorimetric sandwich enzyme-linked immunosorbent assay (ELISA) kits (R&D). Human soluble Aβ_40_ and Aβ_42_ levels were also measured in PRGF-Endoret samples by an ELISA kit (Invitrogen).

### Animals

Male double-transgenic APP/PS1 mice, a cross of the Tg2576 (over-expressing human AβPP695) and mutant PS1 (M146L) mice from our in-house colony (Instituto de Investigacion Hospital 12 de Octubre), were used. Age-matched mice not expressing the transgene were used as wild-type controls. The name of the Institutional Animal Care and Use Committee (IACUC) that approved the study was Comite de Experimentacion y Bienestar Animal. Human Endoret was delivered intranasally 3 times per week for 5 weeks, according to a modified procedure previously described [23]. Mice were briefly anesthetized with isoflurane to ameliorate any suffering and Endoret (total volume of 48 µl) was administered intranasally to APP/PS1 mice, 3 µl at a time, alternating the nostrils, with a lapse of 2 min between each administration, for a total of 16 times. In the control mice, saline (0.9% w/v) was administered. Endoret was administered to 3 and 6 months old APP/PS1 mice groups. From day 8 of the study, 50 mg/kg of BrdU was injected intraperitoneally to each mouse once a day for 7 days, and mice were sacrificed, after deep anesthesia, 28 days later (Figure 1B). All animals were handled and cared for Council Directive 2010/63/UE of 22 September 2010.

### Primary cell culture assays

Primary cortical and hippocampal neurons were obtained from Wistar rat embryos on prenatal day 17 (E17), as previously described [24]. Cultures were kept at 37°C in Neurobasal culture medium (Gibco, Germany) supplemented with 0.5 mM glutamine, 1% antibiotic and 3% B27 in a humidified atmosphere containing 5% CO_2_ for 7 days prior to experimentation. Cell cultures were then incubated in fresh medium with or without Endoret, previously diluted at 7.5% and 10% in a sterile culture medium, alone or in combination with Aβ_42_ (10 µM).

### Immunoassays

#### Western blotting

For western-blot analysis, cell samples were lysed by homogenization with lysis buffer (20 mM Tris-HCl, pH 7.5, 1:1000 Aprotinin, 1:1000 PMSF and 1:1000 Vanadate) and centrifuged for 10 min at 10000 rpm at 4°C. The supernatants were collected and the total protein concentrations were measured by BCA assay (Thermo Scientific, USA). Samples were separated by polyacrylamide gel electrophoresis and transferred to PVDF membranes. After blocking the membranes with 5% dry milk in TTBS for 1 h, membranes were incubated overnight at 4°C with different antibodies in TTBS. The antibodies used included: mouse anti-Hsp70 (1:1000, Santa Cruz Biotechnology), rabbit anti caspase-3 (1:1000, Cell Signaling Technology), and mouse anti-β-actin (1:10000, Sigma). Secondary antibodies were: goat anti-mouse HRP-conjugated (Biorad Laboratories), goat anti-rabbit HRP-conjugated (Biorad Laboratories).

#### Cell death quantification

After 48 h incubation of neuronal primary cultures with Aβ_42_ (10 µM) and 7.5% and 10% Endoret, DNA fragmentation undergoing apoptosis was detected with a Cell Death Detection ELISA^PLUS^ kit (Roche), according to the manufacture’s protocol. In an additional experiment, cell viability 48 h after treatment with Aβ_42_ (10 µM) and PRGF-Endoret was assessed, using the LIVE/DEAD Viability/Cytotoxicity Kit (Molecular Probes). Cell viability was also measured using Cell Counting Kit -8 (CCK-8 assay, Sigma, St. Louis, USA).

### Immunohistochemistry

Mice were deeply anesthetized with isoforane, transcardially perfused with 0.9% saline and brains were immediately removed. Next, tissues were fixed in phosphate-buffered 4% paraformaldehyde, pH 7.4, at 4°C. Fixed brains were cut on a vibratome (Leica Microsystems) at 50 µm, and tissue sections were collected in cold PB 0.1 M, and incubated overnight with primary antibodies at 4 °C. Primary antibodies were: mouse anti-BrdU (1:20000, Hybridoma Bank), rat anti-BrdU (1:400, Chemicon), goat anti- doublecortin (DCX, 1:500, Santa Cruz Biotechnology), mouse anti-NeuN (1:500, Millipore), mouse anti-βIII-Tubulin (1:1000, Promega), rabbit anti-embryonic nerve cell adhesion molecule (ENCAM, 1:500, Millipore) mouse anti-synaptophysin (1:1000, Chemicon), and rabbit anti-synapsin (1:250, Sigma). After overnight incubation, primary antibody staining was revealed using the avidin-biotin complex method (VECTASTAIN Elite ABC Kit, Vector Laboratories, Burlingame, CA) or fluorescence-conjugated secondary antibodies from Molecular Probes.

To estimate the total number of BrdU-positive cells in the brain, we performed DAB staining for BrdU on every sixth brain section. The number of BrdU-positive cells in the granule cell and subgranular cell layer of the dentate gyrus were counted, using light microscopy (Zeiss microscope) at a magnification of 40X, to estimate the total number of BrdU-positive cells in the entire dentate gyrus. Based on a modified stereological method [25], BrdU-positive were counted in one of every six sections from rostral (2 mm from bregma) to caudal (-4.3 mm from bregma). To determine the fate of dividing cells 100-150 BrdU-positive cells across 4-6 sections per mouse were analyzed by confocal microscopy for co-expressing with NeuN. The number of double-positive cells was expressed as a percentage of BrdU-positive cells.

Fluoro-Jade B labeling has been shown to stain degenerated, but not healthy, neurons [26]. Fluoro-Jade B (Histochem, Jefferson, AR) staining was carried out as described previously [27]. Briefly, paraformaldehyde-fixed brain sections were mounted on 1.5% gelatin-coated slides, air-dried overnight at room temperature and then for 30 minutes at 40°C before staining. Sections were immersed for 5 min in a solution containing 1% sodium hydroxide in 80% alcohol, then for 2 minutes in 70% ethanol, and finally for 1 min in distilled water. Sections were then oxidized by immersion for 10 min in 0.06% KMnO4, under moderate shaking. After several rinses in distilled water, sections were incubated for 30 min in 0.004% Fluoro-Jade-B dye in 0.1% acetic acid, rinsed thoroughly in distilled water, and placed into a heater set to 40°C until the tissue was completely dry. Finally, they were cleared in xylene and coverslipped using D.P.X. mounting medium (Sigma). Morphometrical analysis, using ImageJ software (NIH Image), was done as described [20], and results expressed as number of Fluoro-Jade B-positive cells.

### Data and statistical analysis

Results are expressed as means ± standard error of the mean (SEM). Statistical analyses were performed using a two-way ANOVA followed by Tukey’s post hoc test for multiple comparisons. All calculations were made using SPSS v15.0 software. Statistical significance was set at p<0.05.

## Results

### Characterization of Endoret in human samples

As shown in Figure 1, plasma rich in growth factors was obtained following instruction described above (Methods section) from five groups of human subjects (young and old healthy patients, patients with mild cognitive impairment and patients with mild AD and moderate/severe AD) (Table 1), and the levels of some of the most important growth factors were determined (Table 2). We found that IGF-I levels were significantly reduced and HGF levels were increased in elderly non-demented individuals compared with the young group, but no differences were found between age-matched groups. Because platelets contained APP and Aβ peptides [28-32], we investigated levels of Aβ peptides in all the Endoret samples. No significant differences in the concentrations of Aβ_40_ and Aβ_42_ in the Endoret formulations were found between groups (Table 3), suggesting donor suitability of Endoret preparations.

**Table 2:** Concentration of selected proteins and growth factors in human PRGFs samples.

GROUP	**NGF pg/ml**	**VEGF pg/ml**	**IGF-I ng/ml**	**PDGF pg/ml**	**HGF pg/ml**	**TGF ng/ml**
Young	81.65±22.4	130.46± 36.57	95.22±7.98	10.03±2.96	194.5±23.22	13.35±2.23
Aging	49.57±16.8	121.91±32.1	56.15±8.78*	11.76±2.54	308.5±17.3**	9.6±2.3
MCI	71.68±26.9	184.11±56.62	56.93±8.04	5.95±1.26	325.66±36.9	10.7 ±2
AD mild	56.01±28.2	254.41±68.9	52.23±8.1	4.06±0.94	388.84±49.8	9.68±2.02
AD moderate/severe	67.68±25.4	135.36±31.75	66.78±7.64	7.2±1.12	389.41±13.7	12.44±1.87

Data are mean ± SEM; *p<0.05 and **p<0.01 vs young group.

**Table 3:** Concentration of Aß_40_ and Aß_42_ in PRGF-Endoret of donor samples.

GROUPS	**A**ß**_40_ pg/ml**	**A**ß**_42_ pg/ml**
**Young**	6.96±2.93	1.35±0.23
**Aging**	2.91±1.69	2.11±0.61
**MCI**	6.25±4.41	2.94±1.23
**AD mild**	6.66±4.12	1.88±0.49
**AD moderate-severe**	4.8±3.8	1.65±0.38

### Effects of Endoret on proliferation and differentiation in neuronal cell cultures

**Figure 2:**
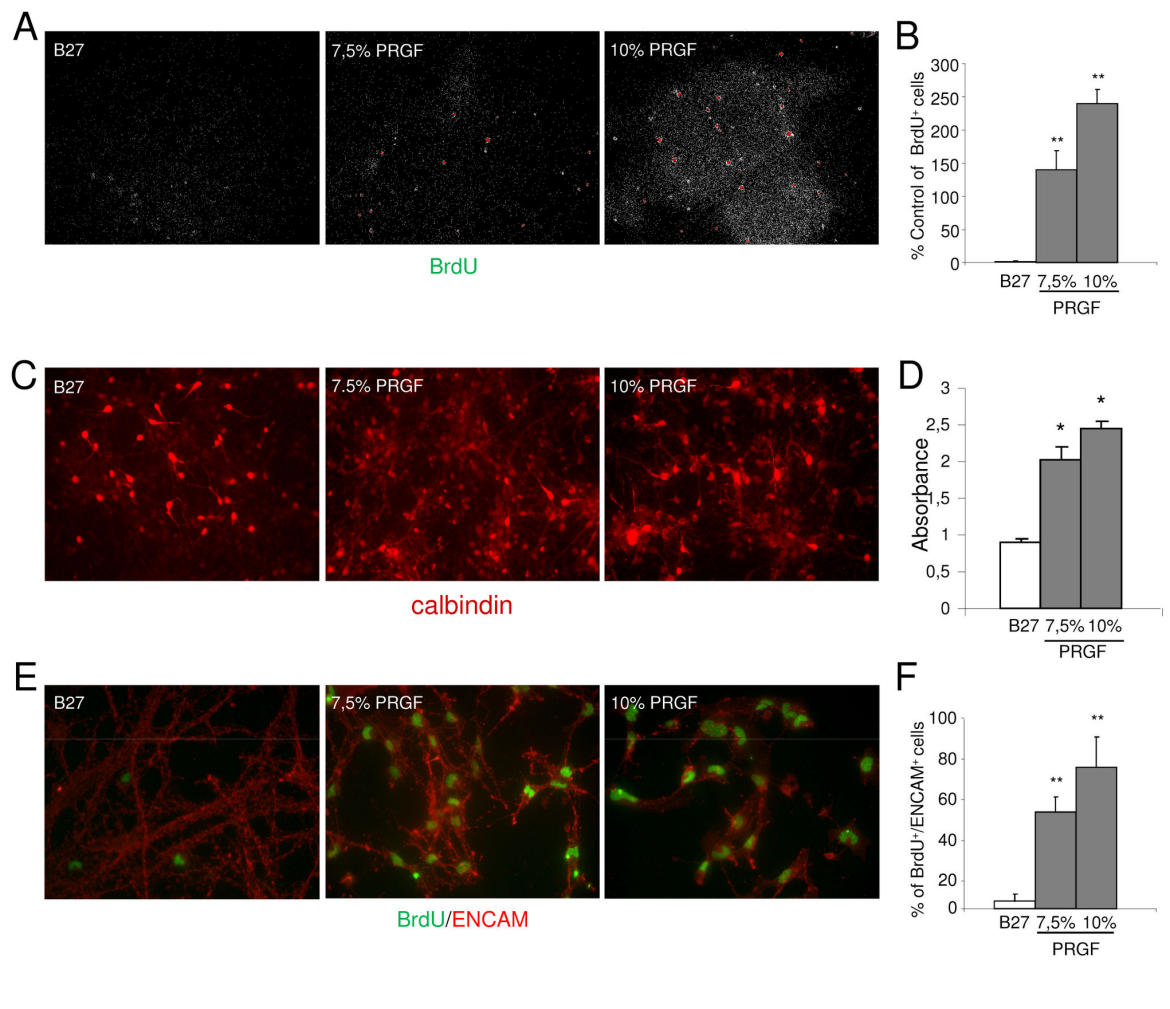
Effects of Endoret on proliferation of primary cultured neurons. A. Fluorescence microscopy images showing BrdU-labeling cells in B27- and Endoret-treated primary cultured neurons. B. Quantitative analysis of BrdU-labeling cells. C Calbindin immunocytochemistry shows an increase in the presence of calbindin-positive neurons in PRGF-treated primary cultured neurons than in B27-treated control group. D. The histogram shows quantitation of calbindin absorbance in each experimental group. E. Representative confocal microscopy images showing co-localization of ENCAM (green) with BrdU (red) in B27- and Endoret-treated primary cultured neurons. F. Quantitative analysis of double ENCAM (green) and BrdU (red)-labeling cells. One representative experiment is shown (n = 3 experiments). Data are mean ± SEM; *p<0.05, **p<0.01 vs control culture.

In a first set of experiments, we used Endoret from the healthy young control group. Because some of the growth factors present in Endoret preparation are involved in modulation of neurogenesis, including IGF-I [20,33], and VEGF [34,35], we explored whether Endoret could play a role in hippocampal neurogenesis. When primary neuronal cells were treated with 7.5% or 10% Endoret for 7 days *in vitro*, an increased incorporation of BrdU into cells was observed (Figure 2A,B). This effect was concentration-dependent and was associated with an increase in cell viability, as demonstrated by calbindin-stained cells (Figure 2C) and XTT absorbance (Figure 2D). Next, we investigated differentiation potential of Endoret analyzing co-localization of neuronal lineage, and we found higher incorporation of BrdU mainly in cells that expressed the immature neuronal marker ENCAM (Figure 2E). Statistical analysis showed the ability of Endoret to increase the number of cell stained with antibodies against BrdU and ENCAM, at concentration of 7.5% and 10% (Figure 2F).

**Figure 3:**
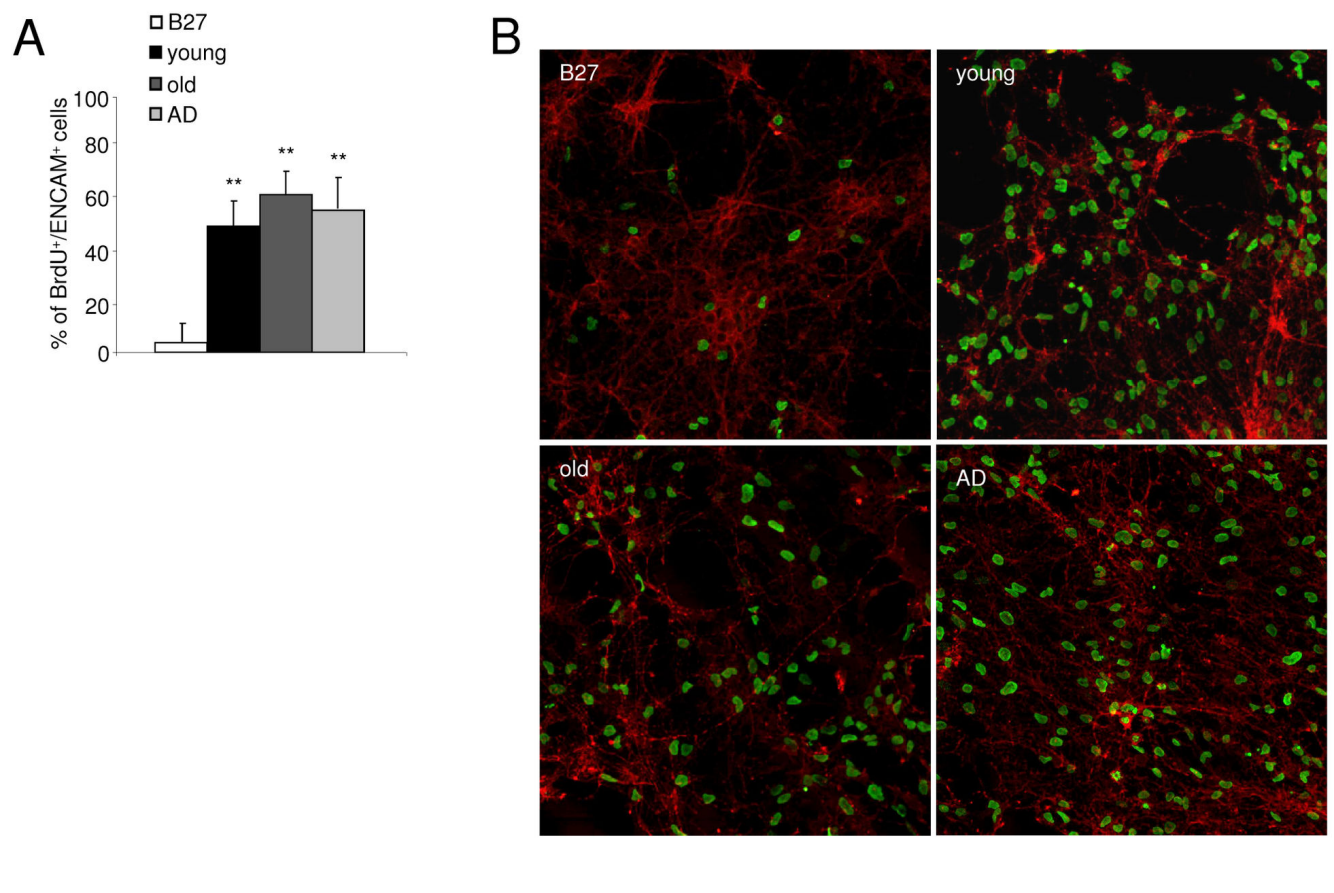
*In vitro* effects of Endoret from young, old and AD patient groups. A. Endoret obtained from different donors (healthy young and old donors and old patients with AD) protects against Ab_1-42_ (10µM)-induced cell death in neuronal cell culture. B. Effects of Endoret on proliferation of primary cultured neurons. Representative confocal microscopy images showing co-localization of ENCAM (green) with BrdU (red) after exposure to 7.5% Endoret from young, elderly, and AD groups. One representative experiment is shown (n = 3 experiments). Data show mean ± SEM; **p<0.01 vs control culture.

Then, we observed that 10% Endoret preparation from young, old and AD patient groups equally enhanced incorporation of BrdU in cultured cells expressing ENCAM (Figure 3A,B), suggesting an efficacy potential of Endoret independently of the donor’s age or health status.

### Protective effects of Endoret against Ab-induced neurotoxicity

**Figure 4:**
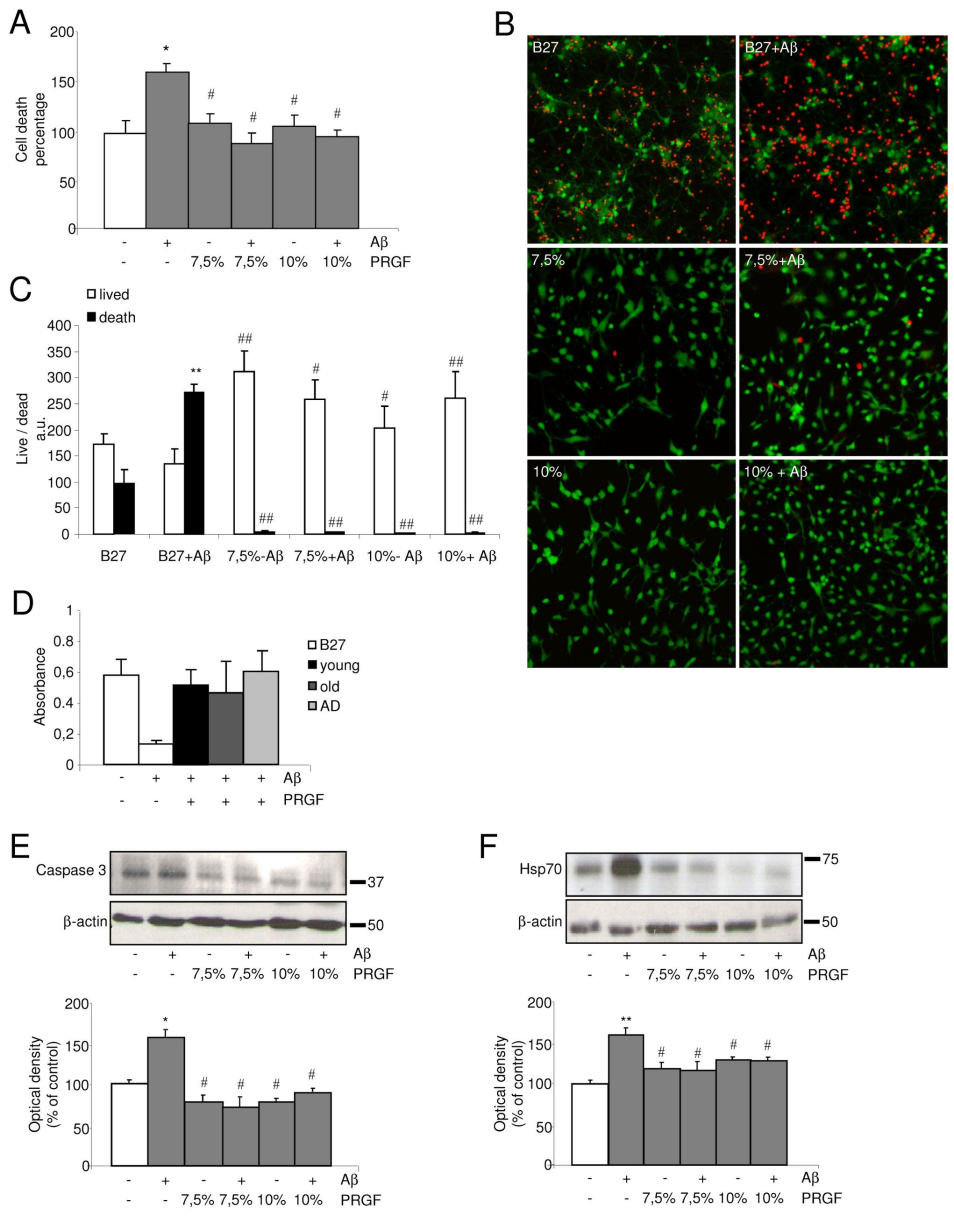
Endoret inhibits apoptotic signaling pathway in Ab-stimulated neuronal cultures. A. 7.5 and 10% Endoret treatment protects against Ab_1-42_ (10 µM)-induced cell death in neuronal cell culture assayed with a cell death ELISA. B. Fluorescent images of living cells (green) and dead cells (red) in neuronal cultures in control condition (B27), and after addition of Ab_1-42_ (10 µM), and 7.5 and 10% Endoret. C. Quantification of Ab_1-42_-induced cell death after 48 h *in* *vitro*. Representative blots and quantitative analysis of (D) caspase-3, and (E) HSP-70 expression in neuronal cultures treated with Aβ_42_ (10 µM) and 7.5 and 10% Endoret. One representative experiment is shown (n = 3 experiments). Data show mean ± SEM; *p<0.05 and **p<0.01 vs control culture. #p<0.05 and # #p<0.01 vs Aβ_42_ -treated culture.

To determine whether amyloidogenic environment could affect cell survival, we investigated the effects of Endoret on neurotoxicity induced by Aβ in primary neuronal cultures. Cultured cells were treated with Aβ_42_ (10 µM) for 48 h. As expected [36,37], there was a significant increase in neuronal death in these cultures treated with Aβ_42_, and this result was completely blocked after co-treatment with 7.5 or 10% Endoret from healthy young control group (Figure 4A). Our findings revealed that modulator effects of Endoret on Aβ_42_-induced neurotoxicity correspond to either reduction of dead neurons but also an increase of live cells (Figure 4B,C). We also tested Endoret from young, old and AD patient groups, and we found that all Endoret preparations prevented Aβ-induced reduction in cell survival in primary neurons (Figure 4D). Western blot analysis performed to assess protein level alterations revealed that this effect in Aβ_42_-induced cell death was preceded by a significant increase in caspase-3 and heat shock protein HSP-70 expression in neuronal culture samples, and co-treatment with PRGF-Endoret was able to block it (Figure 4E,F).

### Endoret enhances hippocampal neurogenesis in APP/PS1 mice

**Figure 5:**
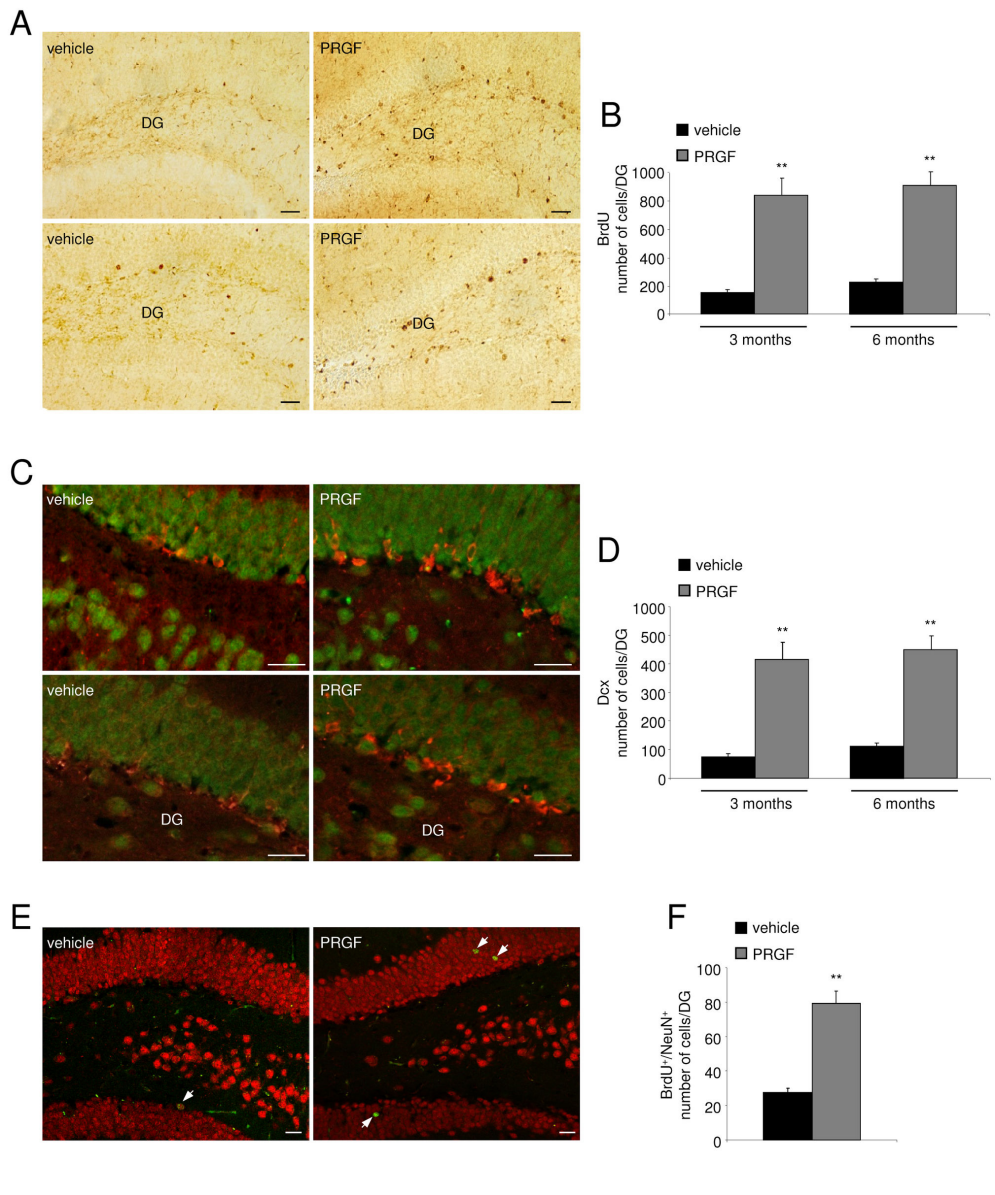
Endoret treatment modulates neurogenesis in APP/PS1 mice. A. Representative fields of BrdU immunostaining in the hippocampal dentate gyrus (DG) of 3 (upper) and 6 months of age (bottom) APP/PS1 mice treated with vehicle or Endoret. Scale bar = 20µm. B. Quantification of BrdU-positive cells after vehicle or Endoret intranasal administration in 3 and 6 month-old APP/PS1 mice. C. Representative confocal microscopy images DG of brain sections immunostained for DCX (red) in combination with NeuN (green) shown in 3 (upper) and 6-month old (bottom) APP/PS1 mice treated with vehicle or Endoret. Scale bar = 20µm. D. Quantification of neurogenesis in 3 and 6 month-old APP/PS1 mice. E. Representative confocal microscopy images DG of brain sections immunostained for BrdU (green) and NeuN (red) in 6 month-old APP/PS1 mice. Arrows point to merged signals. Scale bar = 20µm. F. Quantification of the relative number of BrdU and NeuN double positive cells. n = 9 mice per group. Data show mean ± SEM; **p<0.01 vs APP/PS1 + vehicle.

Finally, we investigate *in vivo* effects of Endoret from healthy young control group using APP/PS1 mice. To determine whether Endoret also stimulates BrdU incorporation in the brain of APP/PS1 mice, Endoret was intranasal administered for 5 weeks, and BrdU (50 mg/kg i.p.) was given daily for 7 days, and brains were examined 4 weeks later. Because a relationship among Ab pathology and hippocampal neurogenesis has been suggested [3,22,25], we used 3 and 6 month-old APP/PS1 mice. Effects of Endoret on cell proliferation were assessed in mouse brain section through the hippocampus, one of the two principal neuroproliferative regions of the adult brain. BrdU-labeled cells were mainly distributed in the inner layer of the granular cell layer of the dentate gyrus (Figure 5A). These BrdU-labeled cells were counted, and we found that there was a significant increase in the number of BrdU-positive cells in the dentate gyrus in both 3 and 6 month-old APP/PS1 mice after Endoret treatment (Figure 5A,B). To ascertain whether Endoret induced the incorporation of BrdU into neurons *in vivo*, as demonstrated above *in vitro*, brain sections from Endoret- and vehicle-treated APP/PS1 mice were processed for double-label immunohistochemistry with antibodies against BrdU and against cell-type-specific markers. The number of DCX-positive newly born neurons in the dentate gyrus in both the early and later stages of the pathogenesis of this mouse model was enhanced by ~400% in Endoret-treated APP/PS1 mice compared with the vehicle-treated group (Figure 5C,D). This length of time (28 days) is known to be sufficient for newly proliferated cells to differentiate into their mature phenotypes. The extent of differentiation of BrdU-labeled cells was determined by double labeling immunohistochemistry with antibodies for BrdU and NeuN (a neuronal marker). Analysis of colocalization of BrdU with NeuN using confocal microscopy indicated that the number of BrdU-labeled cells possessing the neuronal phenotype was significantly increased in 6 month-old APP/PS1 mice with Endoret treatment (Figure 5E,F).

### Endoret reduces neurodegeneration in APP/PS1 mice

**Figure 6:**
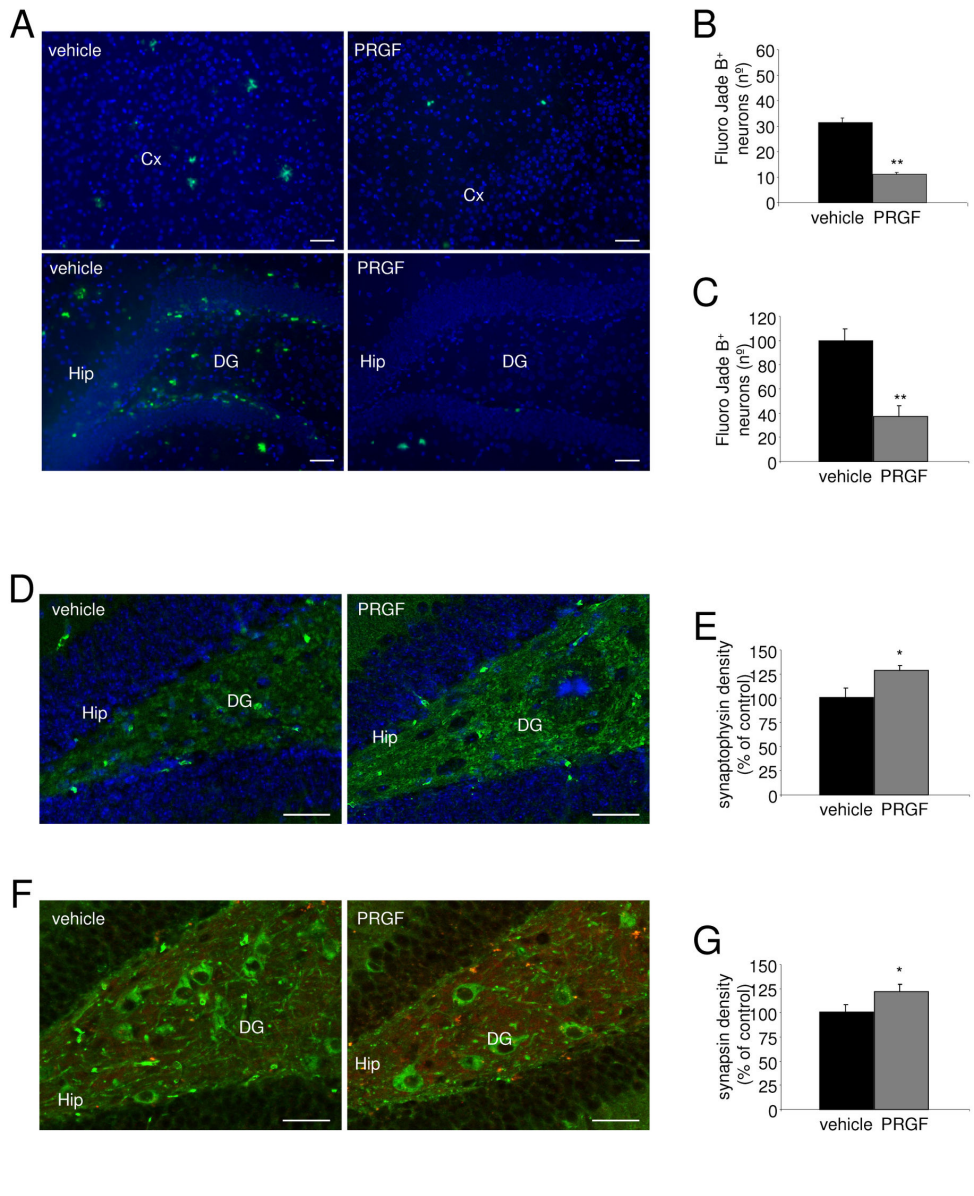
Endoret treatment decreased neurodegeneration in the brain of APP/PS1 mice. A. Representative photomicrographs showing degenerating neurons by Fluoro-Jade B staining in the cerebral cortex (Cx), and hippocampus (Hip) of 6 month-old APP/PS1 mice treated with vehicle and Endoret. Scale bar=20µm. Densitometric analysis of the number (left histogram) and size (in % right histogram) of Fluoro-Jade B-positive plaques in the cerebral frontal cortex (B) and the hippocampus (C) of APP/PS1 mice treated with vehicle and Endoret. D. Representative immunoblots of caspase-3 and synaptic proteins extracted from hippocampal synaptosome preparations. Densitometric quantification of changes in gray values expressed as mean ± SEM (vehicle-treated group, or control, is indicated as 100%). (D) Synaptophysin (green) in combination with DAPI-staining nuclei (blue), and (F) synapsin (red) in combination with bIII-Tubulin (green) labeling in hippocampus of APP/PS1 mice treated with vehicle and PRGF. Quantification of the effect of Endoret administration on synaptophysin (E) and synapin (G) immunoreactivity in hippocampus of APP/PS1 mice. n= 9 mice per group. Data show mean ± SEM; *p<0.05, **p<0.01 vs APP/PS1 + vehicle. Scale bar = 20µm.

Because we have found neuroprotective effects of Endoret on Aβ-induced toxicity in primary neuronal cultures (Figure 4), we examined neuronal degeneration in APP/PS1 mice treated with or without Endoret. Neuronal degeneration was visualized using Fluoro-Jade B staining [27,38], also used as cell death marker [39]. Widespread Fluoro-Jade B-positive neurons were detected in the cerebral cortex and hippocampus of 6 month-old APP/PS1 mice (Figure 6A). Five weeks after treatment with Endoret, Fluoro-Jade B labeling was reduced in the cerebral frontal cortex, and in the hippocampal region in APP/PS1 mice (Figure 6A). Stereological analysis of multiple stained section revealed that the number of Fluoro-Jade B-positive neurodegenerative neurons was reduced in the cerebral cortex (Figure 6B) and the hippocampal dentate gyrus (Figure 6C) of APP/PS1 mice treated with Endoret.

Furthermore, to illustrate the effects of PRGF-Endoret on synaptic markers, synaptophysin, and synapsin were labeled in hippocampus of APP/PS1 mice. Confocal microscopy revealed an increase in these synaptic markers in dentate gyrus of 6 month-old APP/PS1 mice treated with autologous cocktail of proteins (Figure 6D,F). By stereological analysis, we demonstrated that Endoret induced significant recovery of synaptic markers in and dentate gyrus of APP/PS1 mice (Figure 6E,G).

## Discussion

Several recombinant growth factors have been suggested as potential therapeutic agents to prevent or decrease Aβ-associated neurodegeneration. However, the therapeutic role of human plasma and platelet-derived pool of growth factors, delivered by using Endoret technology, in AD pathology has not yet been explored. The main finding of this study is that chronic intranasal Endoret treatment improved neurogenesis and reduced neurodegeneration in APP/PS1 mice.

It is well recognized that new dentate granule cells are continuously generated from neural progenitor cells and are integrated into the existing hippocampal circuitry in the adult mammalian brain through an orchestrated process termed adult neurogenesis [40]. Neurogenesis is regulated by a variety of physiological and pathological stimuli. Increased neurogenesis has been observed in patients with AD, where it could give rise to cells that replace lost neurons [41]. In AD mouse models of amyloidosis, increased hippocampal neurogenesis has also been reported [25,42]. All these findings suggest that stimulating hippocampal neurogenesis could provide a unique approach to AD treatment.

Endoret is an autologous platelet-rich plasma technology by which it is possible to obtain different growth factor-enriched formulations that can be used in the repair and regeneration of a wide range of tissues. The effects of Endoret on tissue regeneration have been demonstrated in dentistry, oral implantology, orthopedics, sports medicine, and treatment of skin disorders [15]. Similarly, the biological effects of plasma and platelet-derived growth factors on the proliferation of various types of cells have been demonstrated [43-45]. However, to our knowledge this is the first report showing potential of Endoret technology on neuronal cells. Although further research is needed to clarify the molecular events that regulate Endoret biological activity, it seems reasonable that some of the proteins present in the autologous cocktail may have played key roles in cell proliferation, differentiation, and survival. Some growth factors present in Endoret preparations have been described as key regulators of neurogenesis, including IGF-I or VEGF [19-22]. Endoret promoted the proliferation and differentiation of neuronal cells in this study, supporting all these previously experimental data. Generating new neurons is a multistep process that includes proliferation, fate choice, migration, survival, and differentiation. We demonstrated for the first time that Endoret promotes proliferation of neuronal progenitors *in vitro*. Although *in vitro* testing is useful, an effective delivery system must be able to mediate successful expression in a relevant *in vivo* model. Stress has been demonstrated to decrease cell proliferation in the dentate gyrus [46]. To avoid these stress-induced effects on neurogenesis, we decided to use an intranasal administration via, which is capable of up taking brain cells, including adult neurons, by entering into the brain, via BBB [47]. Proteins, including growth factors such as insulin or IGF-I, can cross the BBB by an extracellular route along the olfactory bulb or trigeminal neural pathways, as observed by tracing studies with [^125^I]. After intranasal administration, grow factors achieved direct access to the cerebrospinal fluid within 30 minutes, bypassing the bloodstream [48]. Intranasal via system has been shown to efficiently and stably affect brain cells. Drug delivery to the brain via the nasal route is a subject of increasing interest because the nasal mucosa offers rapid absorption with an abundantly vascularized and relatively large absorptive surface area [49], does not require a complicated administration method, and can easily be carried out by medical services for chronic care such as in the case of AD. Growing evidences support this technique in preclinical studies [50-53]. Additionally, numerous clinical trials with Alzheimer’s patients have been successfully performed using intranasal administration of insulin [54-56]. The present data suggest that following intranasal administration of Endoret reached the brain and increased hippocampal neurogenesis.

Another interesting finding herein was the role of Endoret in Aβ-mediated neurotoxicity. Neuroprotection induced by growth factors in AD has been extensively studied [10,11,13,57]. Most of these reports, however, have been focused on the use of a single recombinant growth factor. Additionally, since high doses of recombinant growth factors are needed to achieve biological effects, the risk of systemic side effects would also increases. We have observed that Endoret prevents neuronal death evoked by Aβ, which kills neurons at least partly via inflammatory activation of glia [58]. Endoret also increases the number of live neurons, suggesting that enhanced neuronal survival contributes to neurogenesis in the hippocampus. While it is not completely understood what role the replacement of neurons plays in the brain, it has been suggested that the production of new neurons and their subsequent integration into the neurocircuitry of the brain contribute to cognitive processes including learning and memory [59-61], and could potentially be useful in AD.

We observed that Endoret significantly reduced neuronal degeneration. This result is particularly important because the microenvironment of the AD brain may be toxic to new neurons, and may constitute an important factor in the progression of the neuronal loss, typically observed in patients with AD [62]. This may be one of the reasons why in AD there is a limited repair capacity via neurogenesis. This study demonstrates the extensive trophic actions of Endoret on hippocampal neurons. Our results suggest that in Endoret administration has an important dual action: first, it has a stimulatory effect on neuronal progenitor proliferation, and secondly, it induces a reduction on Aβ-induced neurodegeneration, including in the dentate gyrus, the hippocampal region mainly involved in neurogenesis and memory [63]. These actions also include enhancement of synaptic marker expression. Taken together, these results establish a rationale for Endoret administration as a means for treating hippocampal degeneration in AD.

The significance of neurogenesis in the adult human brain under physiological or pathological conditions is unknown. However, new functional neurons have been shown to arise from the adult human hippocampus, suggesting that nascent neurons in the adult human brain might also have a functional role [64]. We suggest that the increased neurogenesis seen in our experimental model represent an endogenous brain-repair mechanism, the further stimulation of which could have therapeutic potential. Interestingly, and as proof of concept, we also investigated the functional effects of ageing and AD-related Endoret preparations, and similar effects on cell proliferation and survival were observed. These findings suggest that efficacy potential of Endoret may be independent of the donor’s age or health status, representing and autologous biological therapy and an open door to a personalized medicine, though more research is needed to tally confirm these initial evidences.

## Conclusions

In conclusion, Endoret contains high concentrations of a wide array of morphogens, some of them showing a potent stimulating effect on cell proliferation, differentiation and viability. This autologous biological therapy represents a safe and noninvasive therapeutic strategy for the treatment of AD. The possibility of translating these successful results to the prevention and treatment of neurodegenerative disease, including AD, is one of the main medical challenges for the next few years.
